# Introduction to the *Toxins* Special Issue: “Antibodies for Toxins: From Detection to Therapeutics”

**DOI:** 10.3390/toxins14050363

**Published:** 2022-05-23

**Authors:** Eric Ezan, Stéphanie Simon

**Affiliations:** Département Médicaments et Technologies Pour la Santé (DMTS), Université Paris-Saclay, CEA, INRAE, MetaboHUB, F-91191 Gif sur Yvette, France

This Special Issue aims to provide an up-to-date investigation and reviews linked to antibody-based technologies for medical countermeasures and detection/diagnosis tools for toxins. The Toxins Editorial Board has previously edited some Special Issues linked to specific subjects, and we invite readers to join the *Toxins* website for further research in this domain.

Antibodies are key determinants of immune responses. In addition to their role in the host’s fight against infections and intoxications, and thanks to their very high specificity and affinity for their targets, they are a privileged tools for the development of tests and detection methods, and for therapeutic applications.

It is worth recounting benchmarks in this field, and accounting for our debt to researchers who were recipients of the Nobel Prize of Physiology and Medicine for their remarkable work on antibodies. At the end of the 19th century, E. von Behring and K. Shibasaburo proposed the first serotherapy for the treatment of diphtheria and tetanus, for which E. von Behring won the Nobel prize in 1901. Co-winner of the Nobel prize with R. Guillemin and A.W. Schally in 1977, in the 1960s, Rosalyn Yalow, together with S. Berson, established the principle of immunoassays and opened a door to modern clinical chemistry. In 1975, Georges Köhler and César Milstein succeeded in performing fusions of myeloma cell lines with B cells to create immortalized hybridomas, able to produce monoclonal antibodies, receiving the Nobel Prize in 1984. A few years later, the first monoclonal antibodies were licensed for therapeutic and diagnostic applications.

Toxin science has fully benefited from these discoveries, and this *Special Issue* contains 11 publications illustrating recent developments and the use of antibodies for detection, diagnosis, and therapy.

Antibodies are tools of choice for basic research, as they can help us understand the mechanisms of interactions between proteins and between hosts and pathogens, and more generally, they can help us understand the mechanisms induced by a ligand on its target. They are also required for the development of detection tests, in vitro diagnostics, and for therapy. However, the road to the development and marketing of antibodies, in particular for therapeutic applications, is long and full of pitfalls, especially in niche fields where the market cannot be the main driver. In their review, Arnaud Avril and coworkers describe the scientific and industrialization issues encountered in the development of an anthrax therapeutic antibody, which finally led to the discontinuation of the development, even though their humanized antibody had demonstrated good in vitro binding, neutralization capabilities, and promising results in a preclinical model [1].

Upstream of clinical development, an example of antibody characterization for therapeutic applications is given in the article of Delgado et al. [2]. The article describes the development and characterization of therapeutic antibodies directed against ricin (a toxin easy to purify from the plant *Ricinus communis* and potentially used as a biowarfare agent), using in vitro and in vivo (mouse model) approaches. Monoclonal antibodies are an approach of choice for medical countermeasures against ricin intoxication, but they should be thoroughly characterized and selected to recognize different ricin isoforms and cultivars. Their article highlights the need for combined approaches, including adequate testing of affinity, in vitro neutralization, and preclinical models which match real life intoxication. The action mechanisms of antibodies are discussed; these would benefit from being fully characterized, on the one hand to better understand the action mechanisms of the toxin, and on the other hand to better identify the neutralizing capacity of the antibodies themselves. In the same field, Whitfield et al. examined the oral toxicity of ricin in Balb/C mice and developed a robust food deprivation model of ricin oral intoxication that has enabled the assessment of potential antitoxin treatments. Then, they validated ovine F(ab′)_2_ antibody fragments for their protection against aerosolized ricin. Their results demonstrated the benefit of ovine-derived polyclonal antibody antitoxin in providing post-exposure protection against ricin intoxication [3].

Rudenko’s paper is a very good example of the description of an antibody for its therapeutic potential, detection, and as a tool for understanding mechanisms of action. Indeed, Rudenko et al. describe an antibody directed against the hemolysin of *Bacillus cereus*, the fourth most common cause of foodborne illnesses that produces a variety of pore-forming toxins as the main pathogenic factors [4]. The authors presented a panel of monoclonal antibodies to the C-terminal regions of the protein that were usable for detection and found an antibody able to inhibit the pore-forming activity of the toxin. They were able to identify that a leucine in this region is essential in the formation of the pore, causing cell lysis, and that this antibody acts by preventing the oligomerization of the toxin. They discussed the potency of antibodies to recognize various *B. cereus* strains.

Antibodies are necessary tools for detection and clinical diagnosis of toxin intoxication, with a need for the efficient detection and quantification of the toxins in complex matrices, such as environmental, food, and clinical matrices. In this Special Issue, we discuss antibody-based detection and diagnostics tests and their combination with methods, such as mass spectrometry, that can be used for field determination, laboratory confirmation, or forensics. Antibodies thus allow us to provide complementary approaches to the abovementioned techniques and enable us to add an increased level of sensitivity and specificity.

N. Delaunay et al. provide us an extensive review on the interest of using antibodies to immunoconcentrate or purify toxins before liquid chromatography, coupled with different analytical methods. This immunocapture is indeed very useful for selective sample preparation and to limit complex matrix effects, such as food matrices, and to improve the sensitivity of the MS assays: a major breakthrough in the analysis of toxins in recent years. [5]. They also discussed how molecular-imprinted polymers and aptamers might be relevant. Novel instrumentation and commercially available immunosorbents have provided a real improvement in analysis time and the ability to target a wide variety of toxins (from small mycotoxins to larger proteins).

As an example, an immunocapture method combined with mass spectrometry detection (MALDI-Tof) has been developed by Livet et al. for the detection of abrin in food matrices [6]. Abrin is a toxin from *Abrus precatorius*, which is close to ricin in its mechanism of action. Similar to ricin, it has gained considerable interest in the recent past due to its potential misuse as biological warfare agent. Their approach demonstrates how different combinations of immunoaffinity/mass spectrometry can be envisaged with specific advantages in ease of sample preparation, rapidity, specificity, and sensitivity according to the context. The anti-abrin antibodies used for this immunocapture, coupled with mass spectrometry, have been developed by Worbs et al., who used them to develop high-performance ELISAs capable of measuring the toxin in complex matrices (clinical and food) [7]. The developed assays demonstrated their usefulness in the identification of an attempted suicide case involving abrin. Indeed, sandwich ELISAs are methods of choice for measuring and quantifying toxin traces in complex matrices. C. Féraudet-Tarisse et al. have developed and validated immunochromatographic and sandwich ELISA tests for the detection and quantification of staphylococcal toxins, which are very sensitive tests applicable in complex matrices [8]. Staphylococcal food poisoning (SFP) is one of the most common foodborne diseases worldwide, resulting from the ingestion of staphylococcal enterotoxins produced by the bacteria. The authors developed and characterized monoclonal antibodies directed specifically to four enterotoxins and developed sensitive multiplexed immunoassays in different matrices (bacterial cultures of *S. aureus*, contaminated food, and artificially spiked human samples) for different purposes, as follows: strain characterization, food safety, biological threat detection, and diagnosis.

Additionally, Thea Neumann et al. developed very sensitive ELISA tests, based on antibodies and/or a high-affinity cellular receptor (Claudin 4) for the detection and quantification of CPE (enterotoxin produced by *Clostridium perfringens* and responsible for food poisoning and antibiotics-associated diarrhea). With this original approach, they were able to reach sensitivities close to 1.0 pg/mL and were able to detect the CPE in 30 different *C. perfringens* culture supernatants spiked in feces [9].

Sometimes, the targeted toxins are small chemical molecules. In these particular cases, it is impossible to perform sandwich assays, because the molecule is too small to contain two epitopes. It is thus necessary to perform competitive assays, provided that the antigen is easy to label and therefore to detect. The assay then involves a capture antibody, the molecule to be assayed (unlabeled), and the labeled molecule (which serves as a competitor). However, in some cases, the molecule is difficult or impossible to label. It is then possible to use a second antibody, called anti-idiotype antibody, which is supposed to mimic the antigen and compete with it for the binding to the capture antibody. Leivo et al. have implemented this strategy, using a mycotoxin (deoxynivalenol) as a proof-of-concept molecule [10]. They used this molecule and an anti-deoxynivalenol antibody to screen a phage display library and select an anti-idiotype antibody. This antibody allowed the development of a competitive assay with a sensitivity of about 100 ng/mL.

Trying to bring laboratory methods to the field while maintaining the excellent performances of laboratory tests is a major challenge. C. Poehlmann and T. Elssner provided a comprehensive review of commercially available tests and future challenges for the development of antibody-based multiplexed toxin detection platforms. They discussed the needs of first responders for reliable, easy-to-use, and highly sensitive methodologies [11].

Through these 11 publications, this Special Issue shows, once again, the extreme versatility and potentiality of antibodies as essential tools in both basic and applied research. Due to their high selectivity, the first applications have been the design of very sensitive immunoassays, such as ELISA, plasmon resonance assays, lateral flow immunoassays, immunoproteomics, and flow cytometry. In the therapeutic field, antibodies have been used since the 19th century as antitoxins, and remain the tools of choice in the fight against intoxications (bacterial toxins, plant toxins, etc.), for prophylactic and therapeutic purposes.

The field of research involving antibodies is constantly expanding, and has great prospects in the decades to come.
